# Combined short-term exposure to meteorological, pollution factors and pertussis in different groups from Jining, China

**DOI:** 10.7189/jogh.14.04234

**Published:** 2024-10-25

**Authors:** Haoyue Cao, Weiming Hou, Jingjing Jiang, Wenguo Jiang, Xiang Yun, Wenjun Wang, Juxiang Yuan

**Affiliations:** 1 Department of Epidemiology and Health Statistics, School of Public Health, North China University of Science and Technology, Tangshan, Peoples’ Republic of China; 2 Department of medical engineering, Air Force Medical Centre, PLA, Beijing, Peoples’ Republic of China; 3 Infectious Disease Prevention and Control Department, Jining Centre For Disease Control And Prevention, Jining, Peoples’ Republic of China; 4 Department of Nursing, Institute of Public Health Nursing, Weifang Vocational Nursing College, Weifang City, Peoples’ Republic of China

## Abstract

**Background:**

Previous studies have typically explored daily lagged relationships among pertussis and meteorology, with little assessment of effect and interaction among pollutants mixtures.

**Methods:**

Our researchers collected pertussis cases data from 2017–2022 as well as meteorological and contaminative factors for the Jining region. First, we reported the application of the Moving Epidemic Method (MEM) to estimate epidemic threshold and intensity level. Then we developed a Weighted Quantile Sum (WQS) regression and Bayesian Kernel Machine Regression (BKMR) model to assess single, multiple effects and interaction of meteorological and pollution factors on pertussis cases for different sex, delayed and epidemic threshold groups.

**Results:**

There has been a yearly upward trend in the incidence of pertussis in Jining regions. High prevalence threshold years were in 2018–2019, the epidemic peak was mainly concentrated in 32 weeks. Totally, pertussis infections disease was separately 2.1% (95% confidence Interval (CI) = 1.3, 2.8) and 1.1% (95% CI = 0.3, 1.9) higher per decile increase in temperature and sulphur dioxide (SO_2_). And pertussis infections disease was 1.1% lower per decile increase in humidity. In the different stratified analyses, air pressure was a strong negative effect in males and in the lagged 11–20 days group, with 7.3 and 14.7%, respectively. Sulphur dioxide had a relatively weak positive effect in males, females and the group after 20 days lag, ranging from 0.5 to 0.6%. The main positive effectors affecting the onset of disease at low and high threshold levels were ozone (O_3_) and SO_2_, respectively, while the negative effectors were SO_2_ and carbon monoxide (CO), respectively.

**Conclusions:**

This is the first mathematically based study of seasonal threshold of pertussis in China, which allows accurate estimation of epidemic level. Our findings support that short-term exposure to pollutants is the risk factor for pertussis. We should concentrate on pollutants monitoring and effect modeling.

Pertussis is a highly contagious respiratory infection caused by the bacterium *Bordetella pertussis* (*B. pertussis*) and is endemic in all countries worldwide [1]. Existing case definitions include paroxysmal cough, whooping, and posttussive vomiting, but diagnosis can be difficult [2]. Thus, pertussis disease, although well defined, remains endemic worldwide. According to the World Health Organization (WHO) estimated, despite a global immunisation coverage of approximately 86% for pertussis vaccine, there were still 24.1 million cases of pertussis in 2014, with 160 700 deaths occurring in children under the age of five [3]. However, over the past two decades, pertussis has reemerged globally, particularly in countries with high vaccination coverage [4,5]. Pertussis vaccination was introduced in China in the 1950s, and since 2004, the incidence of pertussis has been below 0.5 cases per 100 000 people [6]. However, as in many countries, the reported incidence of pertussis in China has increased in recent years from 0.13 per 100 000 in 2013 to 0.75 per 100 000 in 2017 [7].

Although Zhang et al. [8] have conducted epidemic forecasting research on pertussis using Seasonal Autoregressive Integrated Moving Average (SARIMA) models in the early stage, there is still a lack of threshold calculation and risk level assessment for pertussis during its epidemic period. This is crucial for pertussis epidemic early warning. The occurrence of pertussis is believed to be influenced by a variety of factors, including environmental conditions and climate change. Some researches indicated that pertussis activity was most significantly associated with temperature and rainfall [9]. But little research has been done on the correlation between pertussis and pollutants. Moreover, most studies on infectious diseases and environmental factors have mainly focused on evaluating the impact of individual meteorological or pollution factors on disease occurrence. It is insufficient to demonstrate the long-term effects of overall environmental changes on disease transmission.

The overall goal of this study is to explore the epidemiological characteristics of pertussis infections, graded warnings, single, multiple and interaction effects of climate factors and pollutants. Our specific objectives were to:

a) calculate epidemic thresholds and assess risk levels

b) explore the single and multiple effects of meteorological and pollution factors for different groups

c) analyse the effects and interaction of pollutants mixtures for high and low epidemic level.

## METHODS

### Data collection

We received surveillance data of pertussis infections case from Jining Centre for Disease Control and Prevention for the study area between 2017 and 2022. All patients were diagnosed according to the criteria of pertussis infections diseases’ management issued by the Ministry of Health of the People's Republic of China. We obtained the corresponding daily climate factors and pollutions data from the National Oceanic and Atmospheric Administration including temperature, humidity, inhalable particles (PM_10_), 2.5-µm particulate matter (PM_2.5_), sulphur dioxide (SO_2_) etc.

### Estimation of epidemic threshold and intensity level

In fact, we typically use a certain proportion of pertussis cases as the threshold to define an epidemic. This does not provide insights into the intensity of the epidemic or serve as a prospective indicator for the onset of an epidemic. For respiratory infectious diseases such as pertussis, another method, known as the Moving Epidemic Method (MEM), is widely used to calculate epidemic thresholds [10]. The advantage of this approach is that intensity levels can be calculated and used prospectively [11]. For that, we used the R 4.1.3 (R foundation for Statistical Computing, Vienna, Austria, March, 2022) (package ‘mem’) which is available online for free.

This method is based on a complex mathematical algorithm, which can be summarised into three steps. The first step is to determine the pre-epidemic, epidemic, and post-epidemic phases of the disease. In the second step, the pre and post epidemic values of the historical seasons are used to calculate the baseline and epidemic thresholds. In the third step, select the maximum values of n monitoring indicators during the epidemic period, and calculate different epidemic intensity threshold. The unilateral 50% CI upper limit of the geometric mean of the n maximum surveillance indicators during the epidemic period was defined as the medium intensity threshold; the unilateral 90% CI upper limit as the high intensity threshold; and the unilateral 95% CI upper limit as the very high intensity threshold. The most important parameter for this modelling is δ. We used the ‘roc. analysis’ function to calculate the sensitivity, specificity, Matthew's correlation coefficient and Jordon's index. The best value of δ is filtered by comparing the indices among them. Then, we used the ‘memgoodness’ function to complete the cross-validation, calculate the intensity threshold and define the risk classification.

### Single pollution and Weighted Quantile Sum statistical analyses

Through Spearman correlation analysis, we have preliminarily identified meteorological and pollution factors that are associated with pertussis. Climate factors and pollutants were then incorporated into the Weighted Quantile Sum (WQS) regression model and the effect of the environmental mixture on disease was explored when the overall performance was a positive or negative effect, respectively, and the model can be written as follows: *g(μ) = β_0_+β_1_WQS+z′φ*. Here, *g(μ)* reflects a nonlinear link function allowing generalisation to continuous, binary, and other distributions, though in this study only continuous outcomes were considered. As in typical regression approaches, *β_0_* reflects the model intercept, while *β_1_* reflects the parameter estimate for the co-exposure index, represented here as *WQS*; the significance of this parameter thus reflects a straightforward test of associations between the co-exposure index and outcome [12].

To estimate the combined effects of co-occurring pollutant exposures and evaluate the individual contribution of each factor, we employed a ‘mixture’ approach based on WQS regression analysis. Weighted Quantile Sum regression estimates an index (βWQS) based on empirical data, which helps identify influential exposure variables with non-negligible weights. It allows testing the association between exposure index and outcomes within a traditional linear framework. All analyses in our study were performed in R 4.1.3 (R foundation for Statistical Computing, Vienna, Austria, March, 2022).

### Bayesian kernel machine regression model

We used the Bayesian kernel machine regression (BKMR) model, a non-parametric Bayesian variable selection framework, to evaluate the joint effect of pollutants on pertussis infections disease. BKMR constructs exposure-response functions through iterative regression using Gaussian kernel functions. Due to high correlation among pollutants in the analysis, we employed a Markov Chain Monte Carlo (MCMC) algorithm with 1000 iterations for variable selection. Based on Spearman correlation coefficient values, we separately grouped some pollutants into disease. The formula is as follows:

*Y_i_ = h(z_i1_,…,z_iM_)+β^T^X_i_+e_i_*

where *h()* was the exposure-response function based on nonlinearity and/or interaction among the mixture components, *z_i_* represented pollutants, *X_i_* and β represented covariates and their coefficients, respectively. Confidence intervals obtained from the BKMR model incorporated the additional uncertainty due to estimation of a high-dimension set of exposures and accounting for multiple-testing penalty [13].

### Statistical analysis

We used MEM to estimate the epidemic threshold and intensity level. Then we used Spearman analysis for feature selection in response to the effects of climate and pollution factors. To estimate the individual and combined effects of pollutants exposure, we employed a ‘mixtures’ approach based on WQS regression analysis. Finally, we located the driving factors of pollutants using BKMR model on different epidemic peak of pertussis cases. All analyses in our study were performed in R 4.1.3 (R foundation for Statistical Computing, Vienna, Austria, March, 2022).

## RESULTS

### MEM surveillance for pertussis infections in Jining

**Figure 1:**
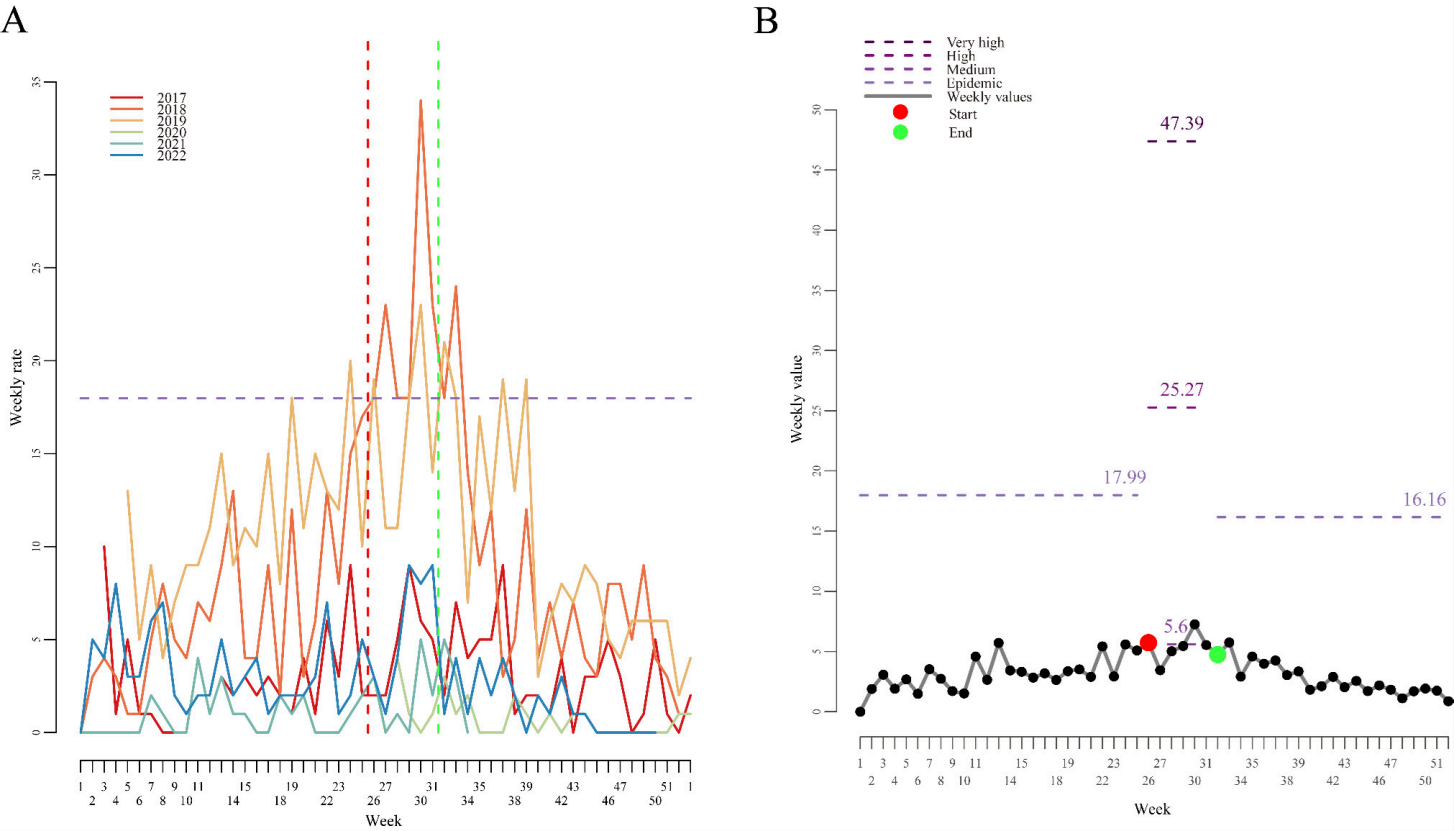
Weekly pertussis-positive proportions (**Panel A**), and moving epidemic model (MEM) epidemic and intensity thresholds (**Panel B**) for 2022/2023 season, Jining, China.

For pertussis infections, there were more male than female patients. The age group was dominated by children aged one–five years, followed by children under the age of one year (mostly in the zero to three months). In the delayed onset and diagnosis group, there were 569 patients (40.85%) mainly in the 11–20 days group. The regional distribution was mainly concentrated in Jining City district, followed by Qufu City. Seasonality is mainly manifested in the high incidence in summer, with patients accounting for about 41.42% (**Table 1**). By the cross-validation process, we identified the optimal parameter for the model as δ = 2.6 from **Table 2** (sensitivity = 0.36, specificity = 0.97). The epidemic threshold was 18.90. By the threshold value, the alert week of the 2022/2023 season was 32th weeks, 2022 (**Table 3**). The peak intensity level was high in 2018/2019–2022/2023, and low in all the other years. By the epidemic threshold value, the alert week of the 2019/2020 season was month two, 2023, which was one month lag for the start time (**Figure 1****,** Panels A–B).

**Table 1:** Distribution of the pertussis cases by sex, age, delay, region and season group in Jining province, 2017–2022

Series	No of whooping cough cases (%)						
	**2017**	**2018**	**2019**	**2020**	**2021**	**2022**	**Total**
**Sex**							
Male	87 (54.72)	238 (50.42)	270 (49.54)	9 (29.03)	26 (61.90)	80 (55.56)	710 (50.97)
Female	72 (45.28)	234 (49.58)	275 (50.46)	22 (70.97)	16 (38.10)	64 (44.44)	683 (49.03)
**Age group**							
0–3 mo	54 (33.96)	83 (17.58)	110 (20.18)	4 (12.90)	13 (30.95)	35 (24.31)	299 (21.46)
4–6 mo	36 (22.64)	78 (16.53)	73 (13.39)	3 (9.68)	10 (23.81)	33 (22.92)	233 (16.73)
7–12 mo	19 (11.95)	86 (18.22)	51 (9.36)	2 (6.45)	3 (7.14)	7 (4.86)	168 (12.06)
1–5 y	39 (24.53)	190 (40.25)	264 (48.44)	18 (58.06)	12 (28.57)	35 (24.31)	558 (40.06)
6–10 y	10 (6.29)	25 (5.30)	32 (5.87)	4 (12.90)	4 (9.52)	33 (22.92)	108 (7.75)
11 y above	1 (0.63)	10 (2.12)	15 (2.75)	0 (0.00)	0 (0.00)	1 (0.69)	27 (1.94)
**Delay group**							
0–10 d	38 (23.90)	141 (29.87)	125 (22.94)	14 (45.16)	16 (38.10)	86 (59.72)	420 (30.15)
11–20 d	64 (40.25)	198 (41.95)	243 (44.59)	10 (32.26)	14 (33.33)	40 (27.78)	569 (40.85)
20 d above	57 (35.85)	133 (28.18)	177 (32.48)	7 (22.58)	12 (28.57)	18 (12.50)	404 (29.00)
**Regions**							
Jining	25 (15.72)	86 (18.22)	130 (23.85)	9 (29.03)	6 (14.29)	40 (27.78)	296 (21.25)
Jiaxiang county	10 (6.29)	44 (9.32)	23 (4.22)	1 (3.23)	8 (19.05)	11 (7.64)	97 (6.96)
Jinxiang county	4 (2.52)	35 (7.42)	56 (10.28)	0 (0.00)	3 (7.14)	15 (10.42)	113 (8.11)
Liangshan county	16 (10.06)	69 (14.62)	73 (13.39)	1 (3.23)	5 (11.90)	9 (6.25)	173 (12.42)
Qufu city	24 (15.09)	101 (21.40)	94 (17.25)	12 (38.71)	3 (7.14)	13 (9.03)	247 (17.73)
Sishui county	33 (20.75)	25 (5.30)	38 (6.97)	1 (3.23)	0 (0.00)	13 (9.03)	110 (7.90)
Weishan county	6 (3.77)	33 (6.99)	21 (3.85)	1 (3.23)	8 (19.05)	5 (3.47)	74 (5.31)
Wenshang county	8 (5.03)	22 (4.66)	26 (4.77)	0 (0.00)	4 (9.52)	15 (10.42)	75 (5.38)
Yutai county	10 (6.29)	26 (5.51)	36 (6.61)	0 (0.00)	3 (7.14)	8 (5.56)	83 (5.96)
Zoucheng city	23 (14.47)	31 (6.57)	48 (8.81)	6 (19.35)	2 (4.76)	15 (10.42)	125 (8.97)
**Seasons**							
Spring	36 (22.64)	88 (18.64)	169 (31.01)	6 (19.35)	1 (2.38)	41 (28.47)	341 (24.48)
Summer	63 (39.62)	236 (50.00)	206 (37.80)	5 (16.13)	13 (30.95)	54 (37.50)	577 (41.42)
Autumn	36 (22.64)	88 (18.64)	85 (15.60)	7 (22.58)	12 (28.57)	26 (18.06)	254 (18.23)
Winter	24 (15.09)	60 (12.71)	85 (15.60)	13 (41.94)	16 (38.10)	23 (15.97)	221 (15.87)
Summary	159	472	545	31	42	144	1393

**Table 2:** Screening results of parameter δ value

δ value	Sensitivity	Specificity	PPV	NPV	MCC	YI
2.0	0.44	0.94	0.58	0.90	0.42	0.38
2.1	0.44	0.94	0.58	0.90	0.42	0.38
2.2	0.44	0.94	0.58	0.90	0.42	0.38
2.3	0.42	0.95	0.61	0.90	0.43	0.37
2.4	0.42	0.95	0.61	0.90	0.43	0.37
2.5	0.38	0.95	0.58	0.89	0.40	0.33
2.6	0.36	0.97	0.70	0.89	0.44	0.33
2.7	0.36	0.97	0.70	0.89	0.44	0.33
2.8	0.36	0.97	0.70	0.89	0.44	0.33
2.9	0.36	0.97	0.70	0.89	0.44	0.33
3.0	0.36	0.97	0.70	0.89	0.44	0.33
PPV – positive predictive value, NPV – negative predictive value, MCC – Matthews correlation coefficient, YI – Youdens index						

**Table 3:** Characteristics of peak values in each year used in model

Year	Peak	Peak week	Epidemic threshold	Threshold intensity			Level
				**Medium**	**High**	**Very high**	
2017	10.00	1	18.73	18.73	28.49	57.28	Baseline
2018	34.00	31	17.54	17.54	16.79	29.91	Very high
2019	23.00	26	12.50	12.50	20.44	38.26	High
2020	4.00	1	17.52	17.52	28.04	49.33	Baseline
2021	5.00	49	18.28	18.28	29.75	60.10	Baseline
2022	9.00	32	18.90	18.90	26.68	52.27	Baseline

### Environmental factors exploration using Spearman and WQS regression model analysis

**Figure 2:**
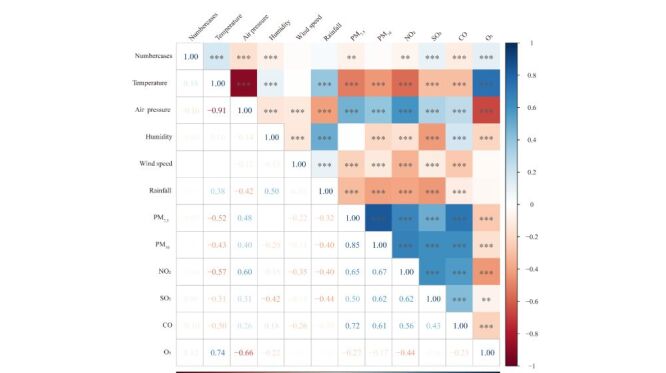
Correlation map of the pollutants and climate factors. Heat Map shows the Spearman correlation coefficients (r) between each environmental factor.

Spearman correlation analysis showed that the cases of pertussis infections were significantly correlated with some pollutants and climate factors (**Figure 2**). In total, WQS index was positively associated with pertussis infections cases (odds ratio (OR) = 0.52; 95% confidence interval (CI) = 0.40, 0.64) in the positive direction of pertussis infections. By contrast, WQS index was negatively associated with pertussis infections cases (OR = −0.43; 95% CI = −0.54, −0.31) in the negative direction of pertussis infections (**Table 4**). The stratified analysis yielded similar effect directions to the overall analysis, except that the group with an 11–20 days lag in onset of symptoms showed no positive effect.

**Table 4:** Association of pollutions and climate factors with pertussis – stratified by sex and lagging group

Series	Direction	β_WQS_ (95% CI)	*P*-value
Total	Positive	0.52 (0.40, 0.64)	<0.001
	Negative	−0.43 (−0.54, −0.31)	
Male	Positive	0.27 (0.20, 0.35)	
	Negative	−0.23 (−0.30, −0.16)	
Female	Positive	0.24 (0.18, 0.31)	
	Negative	−0.20 (−0.26, −0.13)	
0–10 d	Positive	0.10 (0.04, 0.15)	<0.01
	Negative	−0.06 (−0.10, −0.02)	
11–20 d	Positive	n/a	n/a
	Negative	−0.23 (−0.30, −0.16)	<0.001
20 d above	Positive	0.20 (0.14, 0.27)	
	Negative	−0.16 (−0.21, −0.11)	

CI – confidence interval, n/a – not applicable, WQS – Weighted Quantile Sum

**Figure 3:**
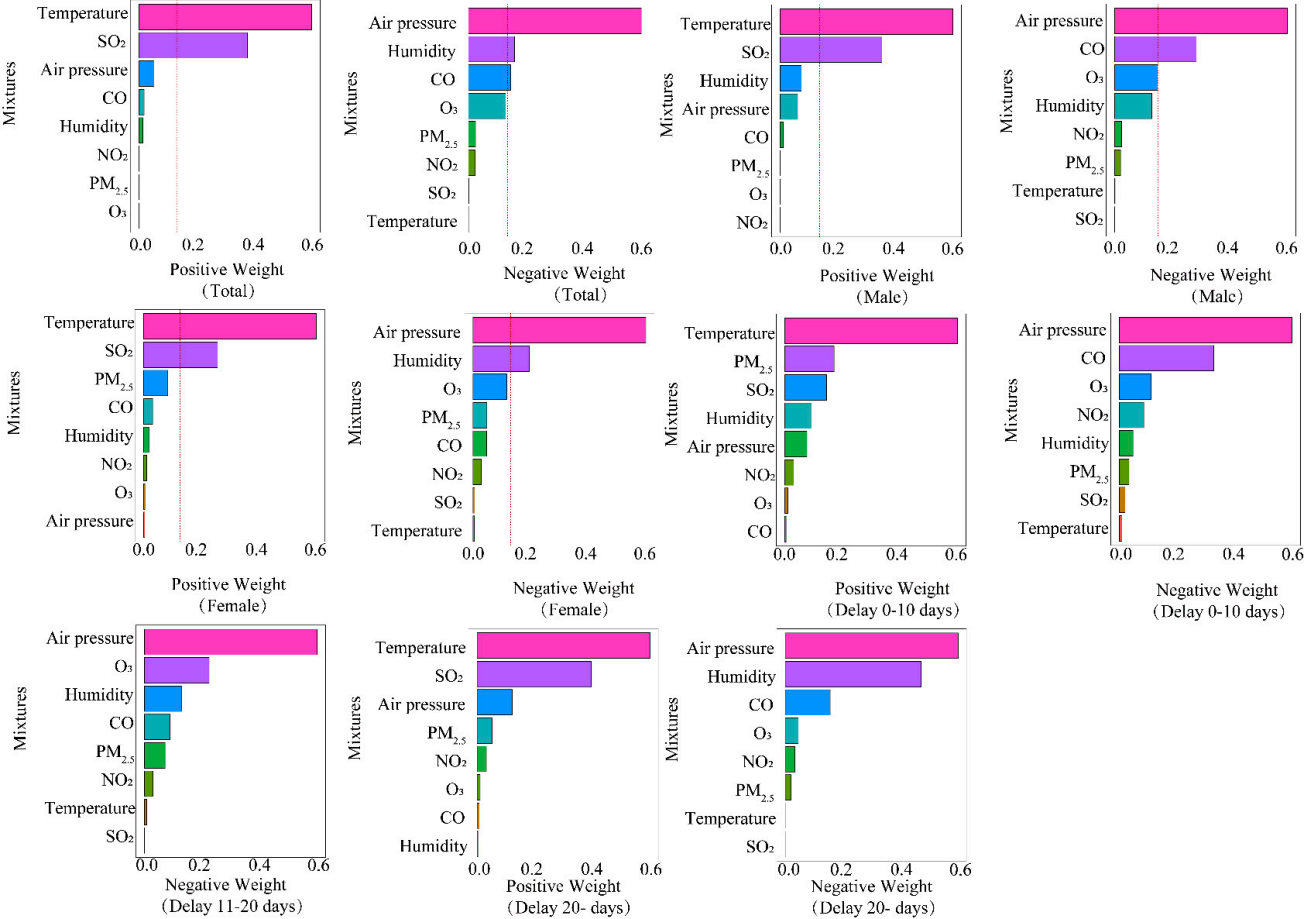
Weighted Quantile Sum (WQS) model regression index positive and negative weights for total, sex and delayed group. Models were adjusted for age.

Totally, pollutants and climate factors with the highest positive estimated weights for cases outcomes were temperature (WQS weight = 0.56) and SO_2_ (WQS weight = 0.35). Pollutants and climate factors with the highest negative estimated weights for cases outcomes were air pressure (WQS weight = 0.55) and humidity (WQS weight = 0.15) (**Figure 3**). Sex and onset lag stratification groups were generally like the overall. However, in the male and delay zero–ten days groups, the highest negative estimated weights for case outcomes included carbon monoxide (CO). In the zero–ten days lag group, the highest positive estimated weight for the case outcome includes PM_2.5_.

### Assess the association among climate factors, pollutants, and pertussis infections cases for different groups

Totally, survey-weighted single pollution analyses indicated that pertussis infections disease was separately 2.1% (95% CI = 1.3, 2.8) and 1.1% (95% CI = 0.3, 1.9) higher per decile increase in temperature and SO_2_. Pertussis infections disease was 1.1% lower per decile increase in humidity (**Table 5**). Through environmental mixture analysis of the factors preselected by WQS regression, we found that overall temperature and SO_2_ have a positive impact on the incidence rate, with an effect size of 0.38 (95% CI = 0.29, 0.46). On the other hand, air pressure, humidity, and CO have a negative impact on the incidence rate, with an effect size of −0.34 (95% CI = −0.43, −0.25).

**Table 5:** Comparison of results from the survey-weighted single pollution analyses and Weighted Quantile Sum (WQS) regression of the matrix specific pollutions mixtures for the pertussis

Series	Direction	Mixtures	Single pollution regression survey-weighted		Multiple pollution regression survey-weighted	
			**β_WQS_ (95%CI)**	***P*-value**	**β_WQS_ (95% CI)**	*P*-value*
Total	Positive	Temperature	0.021 (0.013, 0.028)	<0.001*	0.38 (0.29, 0.46)	<0.001
		SO_2_	0.011 (0.003, 0.019)	<0.01*		
	Negative	Air pressure	−0.080 (−0.197, 0.037)	0.178745	−0.34 (−0.43, −0.25)	<0.001
		Humidity	−0.011 (−0.016, −0.005)	<0.001*		
		CO	0.046 (-0.078, 0.171)	0.468		
Male	Positive	Temperature	0.015 (0.011, 0.019)	<0.001*	0.19 (0.14, 0.25)	<0.001
		SO_2_	0.005 (0.000, 0.010)	<0.05*		
	Negative	Air pressure	−0.073 (−0.137, −0.009)	<0.05*	−0.19 (−0.25, −0.13)	<0.001
		CO	0.030 (−0.047, 0.108)	0.44		
		O_3_	0.001 (0.000, 0.002)	<0.05*		
Female	Positive	Temperature	−0.001 (−0.007, 0.005)	0.784	0.18 (0.13, 0.23)	<0.001
		SO_2_	0.006 (0.002, 0.011)	<0.01*		
	Negative	Air pressure	−0.077 (−0.172, 0.018)	0.1114	−0.11 (−0.16, −0.07)	<0.001
		CO	−0.001 (−0.070, 0.067)	0.969		
0-10 d	Positive	Temperature	0.004 (0.001, 0.007)	<0.01*	0.06 (0.03, 0.09)	<0.001
		PM_2.5_	0.0002 (−0.001, 0.001)	0.574389		
		SO_2_	0.001 (−0.002, 0.004)	0.339556		
	Negative	Air pressure	−0.013 (−0.058, 0.032)	0.57	−0.05 (−0.08, −0.02)	<0.01
		CO	0.021 (−0.025, 0.068)	0.370287		
11-20 d	Negative	Air pressure	−0.147 (−0.219, −0.075)	<0.001*	−0.16 (−0.21, −0.11)	<0.001
		O_3_	0.001 (0.000, 0.002)	0.105		
20 d above	Positive	Temperature	−0.002 (−0.006, 0.002)	0.361	0.16 (0.12, 0.21)	<0.001
		SO_2_	0.005 (0.002, 0.009)	<0.01*		
	Negative	Air pressure	−0.034 (−0.094, 0.026)	0.271	−0.08 (−0.11, −0.05)	<0.001
		CO	0.008 (−0.048, 0.063)	0.788		

*Significant results.

CI – confidence interval, CO – carbon monoxide, O_3_ – ozone, PM_2.5_ – particulate matter, SO2 – sulphur dioxide, WQS – Weighted Quantile Sum

In the different stratified analyses, air pressure was a strong negative effect in males and in the lagged 11–20 days group, with 7.3 and 14.7%, respectively. SO_2_ had a relatively weak positive effect in males, females and the group after 20 days lag, ranging from 0.5 to 0.6% (**Table 5**).

### Analyse the effects and interaction of pollutants mixtures for high and low epidemic level

**Figure 4:**
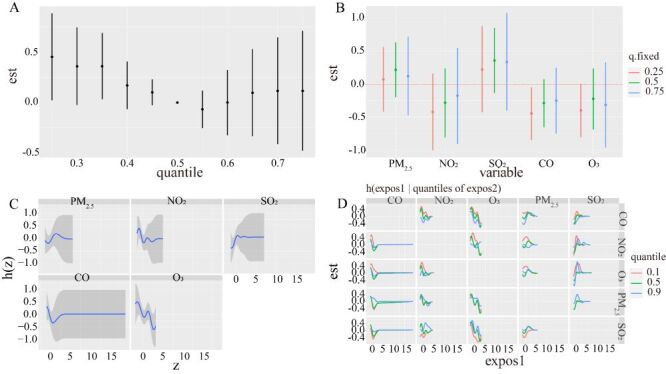
Associations between pollutants mixtures and pertussis disease among the study population by Bayesian Kernel Machine Regression (BKMR)model. Model adjust for climate indicators by using Spearman correlation. **Panel A.** The cumulative effect of the pollutant’s mixtures (estimates and 95% credible intervals). Pollutants mixtures are at a particular percentile (x-axis) compared to when exposures are all at 50th percentile. **Panel B.** The single-exposure effect (estimates and 95% credible intervals). **Panel C.** Univariate exposure-response functions and 95% confidence bands for each pollutant with the other mixtures fixed at the median. **Panel D.** Multiple exposure-response functions for: the other pollutant when one metal fixed at either the 25th, 50th, or 75th percentile and the test of pollutants mixtures is fixed at the median.

We show the visualisation of the BKMR model. It was seen that the total mixtures effect of pertussis infections tended to decrease and then increase with increasing exposure time. In particular, when the mixture of all pollutants is between the 10th and 30th percentiles, we found that the overall effect is statistically significant (**Figure 4**, Panel A). Single effect showed that CO and O_3_ concentration in 25th percentile is associated with a significant decrease in pertussis infections of -0.445 (−0.846, −0.045) and −0.400 (−0.803, −0.005) (**Figure 4**, Panel B**)**. To investigate potential nonlinearity of the exposure-response function, we then estimated the univariate relationship between each pollutant and cases, where all the rest of pollutants are fixed to 50th percentile. 2.5-µm particulate matter and SO_2_ show an inverted U-shaped relationship with pertussis infections cases instead of CO (**Figure 4**, Panel C). To explore potential the relationship between pollutants mixtures further, we plotted bivariate cross-sections of exposure-response function. **Figure 4**, Panel D shows differences in pertussis infections disease as a function of Nitrogen Dioxide (NO_2_), by moving ozone (O_3_) and SO_2_ concentrations from 25th to 50th and to 75th percentile (while fixing all other pollutants mixtures to their median).

**Figure 5:**
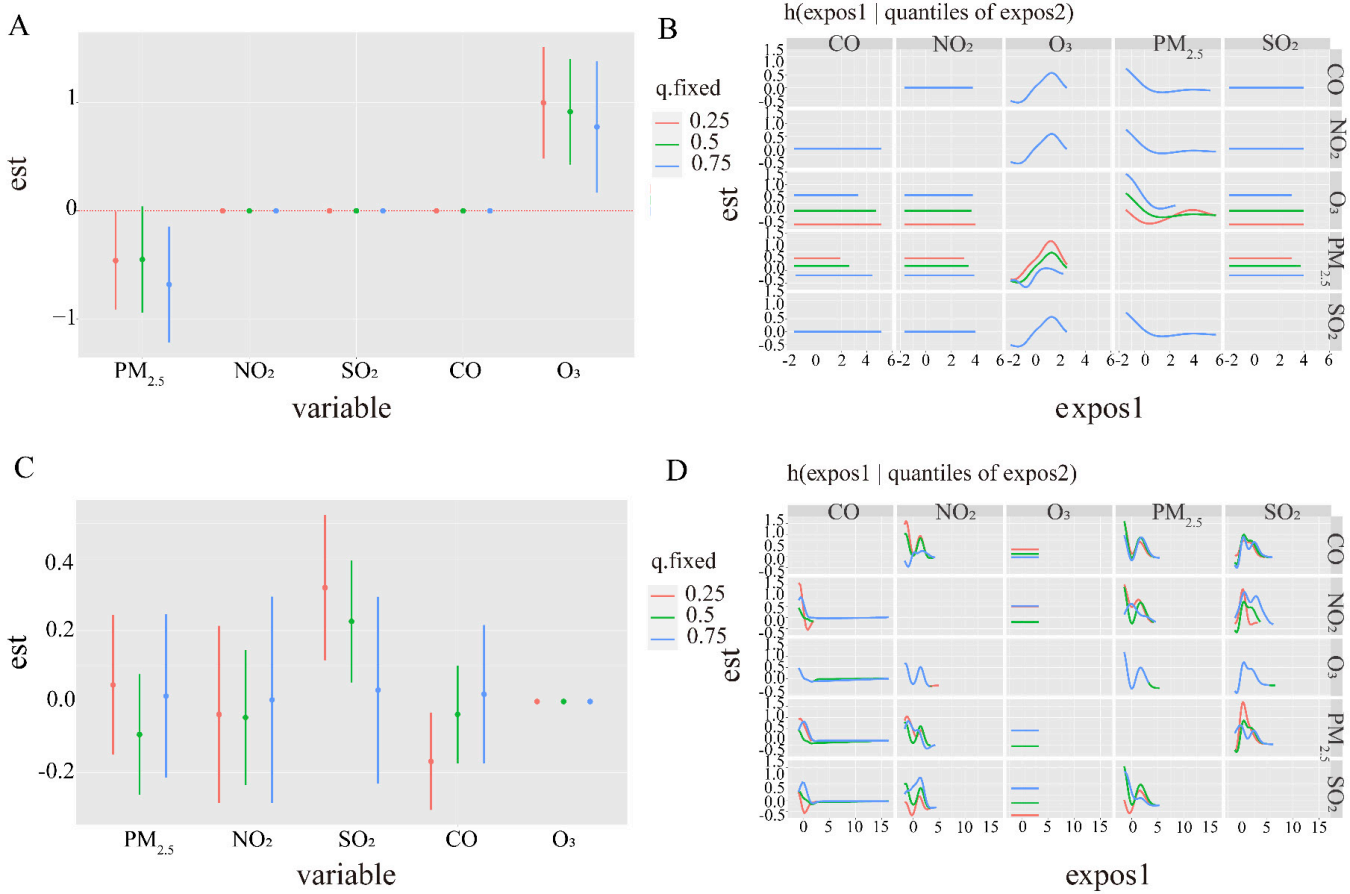
Associations between pollutants mixtures and pertussis disease among the study population by Bayesian Kernel Machine Regression (BKMR) model for high and low epidemic threshold. Model adjust for climate indicators by using Spearman correlation. **Panel A.** The single-exposure effect for high level (estimates and 95% credible intervals). **Panel B.** Multiple exposure-response functions for: the other pollutant when one metal fixed at either the 25th, 50th, or 75th percentile and the test of pollutants mixtures is fixed at the median for high level. **Panel C.** The single-exposure effect for low level (estimates and 95% credible intervals). **Panel D.** Multiple exposure-response functions for: the other pollutant when one metal fixed at either the 25th, 50th, or 75th percentile and the test of pollutants mixtures is fixed at the median for low level.

From **Figure 5**, Panel A, PM_2.5_, and O_3_ in pollutants mixtures under low epidemic threshold were associated with pertussis infections cases, with negative and positive effects, respectively. From **Figure 5**, Panel B, only PM_2.5_ and O_3_ interacted during the low threshold period. The main environmental mixtures with effects at the high epidemic threshold include SO_2_ and CO, with positive and negative effects, respectively (**Figure 5**, Panel C). From **Figure 5**, Panel D, both SO_2_ and NO_2_ interacted with other pollutants on pertussis infections disease.

## DISCUSSION

Although there have been numerous predictive studies on pertussis conducted both domestically and internationally, from SARIMA models to Nonlinear Autoregressive Network have used time series analysis, and have achieved good results [14]. However, for such respiratory infectious diseases, single epidemic predictions alone are already insufficient to meet the needs of future epidemiology. Currently, we need to monitor epidemics prospectively and in near-real-time, as well as issue warnings about diseases. So, we need a more detailed method that can calculate its epidemic threshold and evaluate the intensity of its epidemic to address this deficiency. In the European Centre for Disease Control and Prevention, the MEM method is a standardised method for epidemiological classification and early warning of infectious diseases [15]. Min Kang and colleagues [16] used this method to calculate the epidemic threshold for influenza in Guangdong Province, China, and determined the activity level for each season based on this threshold. The Netherlands has also evaluated the seasonality of Respiratory Syncytial Virus using the MEM method and has achieved promising progressive results [11]. Based on this, we can conclude that the MEM method is simpler compared to traditional predictive models and has better warning effectiveness. In the future, this method should be advocated for infectious disease surveillance and early warning.

This study provides an excellent methodology for the exploration of epidemiological trends in pertussis disease, and is considerably more precise and convincing than previous descriptive analyses [17].

Several studies have examined whether individual meteorological and air pollution exposures are associated with pertussis [18]. However, few studies have quantitatively analysed pertussis and environmental factors. The present study found a positive effect of temperature and SO_2_ on incidence. Higher temperatures increased the sensitivity of the pertussis vaccine, causing it to change in some way. Also, in the epidemic summer, high temperatures increase children's exposure to environmental pollutants, which can keep diseases and their transmission at an epidemic level [19]. This finding is consistent with a survey that showed both tuberculosis and pertussis are closely associated with total smoke and SO_2_ emissions [20]. In recent years, haze pollution has attracted much attention due to its significant impact on public health [21]. In contrast, air pressure, humidity and CO have a negative effect on morbidity. Previous studies have found that the higher the humidity, the faster the growth of *B. pertussis* on agar plates. In addition, humid environments increase the number of pathogens deposited on the surface of objects, thus increasing the chance of infection [22,23]. The reason for the opposite results may be due to the fact that the study was conducted in the Shandong Peninsula in China, where the climate is more humid compared to the inland areas at the same latitude. So, residents were reluctant to go out in the summer months, and children were exposed to poor indoor air circulation in a room with low relative humidity, which increased their chances of getting sick. The lower outdoor CO concentration was also due to the reduced use of coal during the summer. This, to some extent, led children to overlook outdoor pollution and increased their opportunities for exposure to bacteria.

This study employed a dual-layer and stratified approach in environmental modeling strategy. We conducted single and mixed pollution analysis, and compared the similarities and differences of single and mixed meteorological and pollution effects under different genders and different initial lag conditions. We found little difference between males and females, but males showed significant significance in the negative CO effect. This could be because boys tend to prefer outdoor activities, making them more likely to increase exposure to pathogenic organisms under low concentrations of carbon monoxide [24]. In contrast, the positive effect of PM_2.5_ was significantly significant in the delay zero to 10 days, which was similar to the results of a Chinese survey [20]. Within these circumstances, we also explored the overall and interactive effects of pollutants on whooping cough incidence in Jining City, Shandong Province, China, at different prevalence thresholds. At low threshold and high threshold levels, the main positive effect factors influencing the onset of the disease were O_3_ and SO_2_, while the negative effect factors were SO_2_ and CO. This will provide us with a basis for future monitoring of different contaminants in response to epidemics of pertussis disease, depending on the period of epidemic level.

Although the associations of pertussis with pollutants and meteorological factors were analysed in depth in this study, the findings could not be extrapolated to all regions of China due to regional limitations. Moreover, there was a significant lag between the onset and diagnosis of pertussis in this study, the lagged stratified analysis of environmental factors such as pollutants still leads to uncertainty in the effects of environmental factors. Thus, it could pave the way for future researchers to explore their associations.

## CONCLUSIONS

This study calculated the seasonal threshold of pertussis based on an epidemiological model. This could accurately estimate the epidemic level of the disease. Short-term exposure to air pollutants such as SO_2_ and O_3_ could increase the risk of pertussis incidence. Different genders and diagnostic delay days had different sensitivities to various pollutants. In the future, during periods of high pertussis prevalence, it is important to focus on monitoring susceptible populations to prevent the spread of pertussis. Additionally, strengthening meteorological monitoring and management of pollutant emissions should be emphasised.
